# Supplementary figures and images for: Nucleus Accumbens Dopamine Encodes the Trace Period during Appetitive Pavlovian Conditioning
Source: eNeuro. 2025 May 20;12(5):ENEURO.0016-25.2025. doi: 10.1523/ENEURO.0016-25.2025 (PMC12113932; doi:10.1523/ENEURO.0016-25.2025)

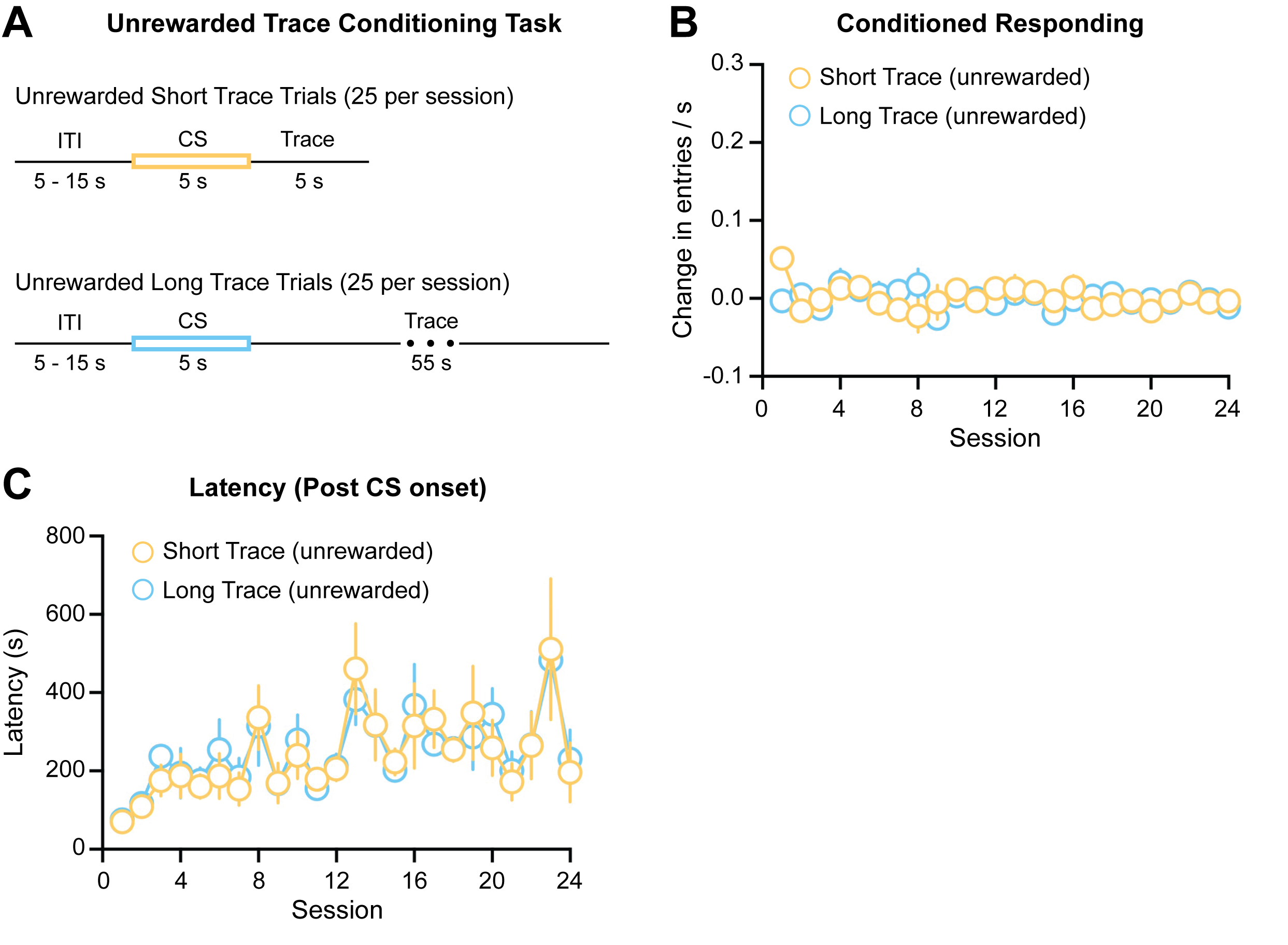

Supplement: Figure 1-1 — Unrewarded trace conditioning task. (A) Schematic of the Unrewarded Short and Long Trace trials, which are presented in a pseudorandom pattern within a session. (B) No change in conditioned responding to unrewarded cues across training sessions (N = 5 rats; 2 male and 3 female). (C) Response latency following the CS presentation across training sessions. Download Figure 1-1, TIF file. [file eneuro-12-ENEURO.0016-25.2025-s001.tif]

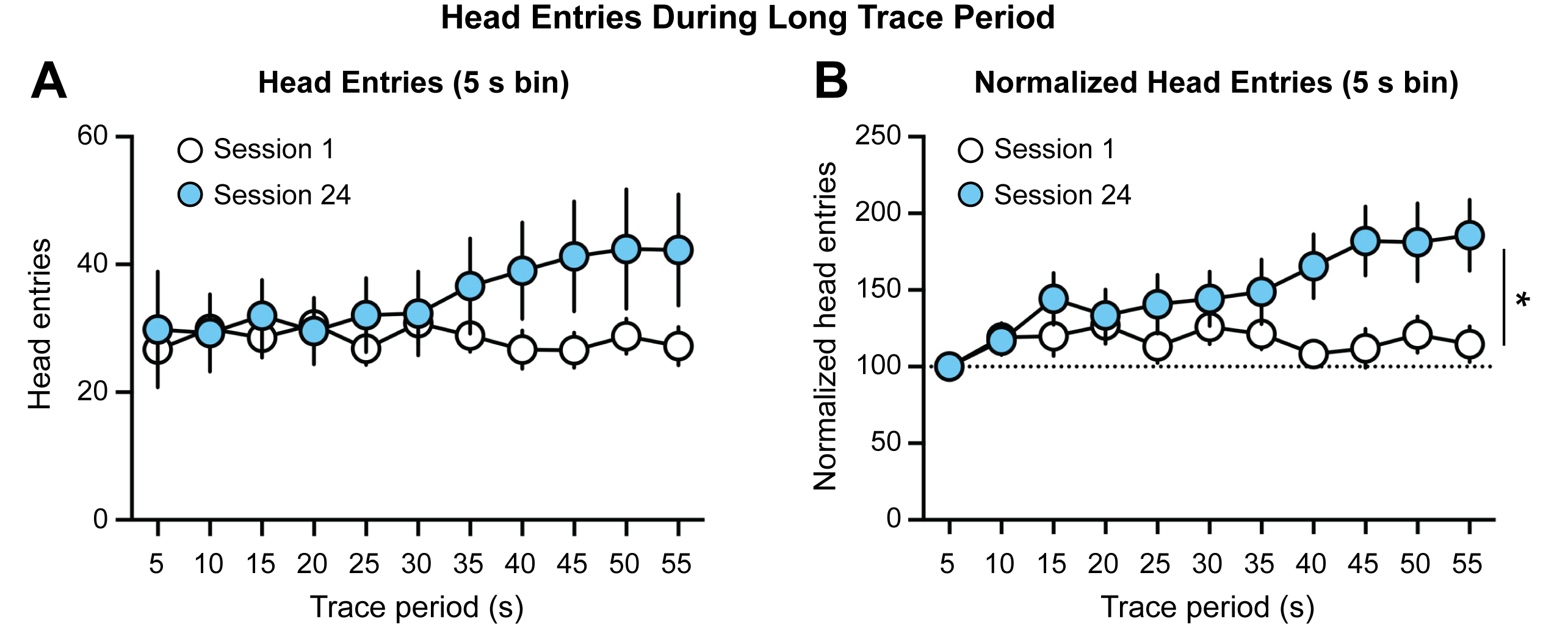

Supplement: Figure 1-2 — Head entries during the long trace period. (A) Head entries increase throughout the trace period over training (two-way ANOVA: interaction effect F(10,220) = 4.4, p < 0.001). (B) Head entry data normalized to the responding during the first 5 s of the trace period illustrates an increase in responding throughout the trace period over training (two-way ANOVA: interaction effect F(10,220) = 4.6, p < 0.001; training effect F(1,22) = 6.6, * p = 0.02). Download Figure 1-2, TIF file. [file eneuro-12-ENEURO.0016-25.2025-s002.tif]

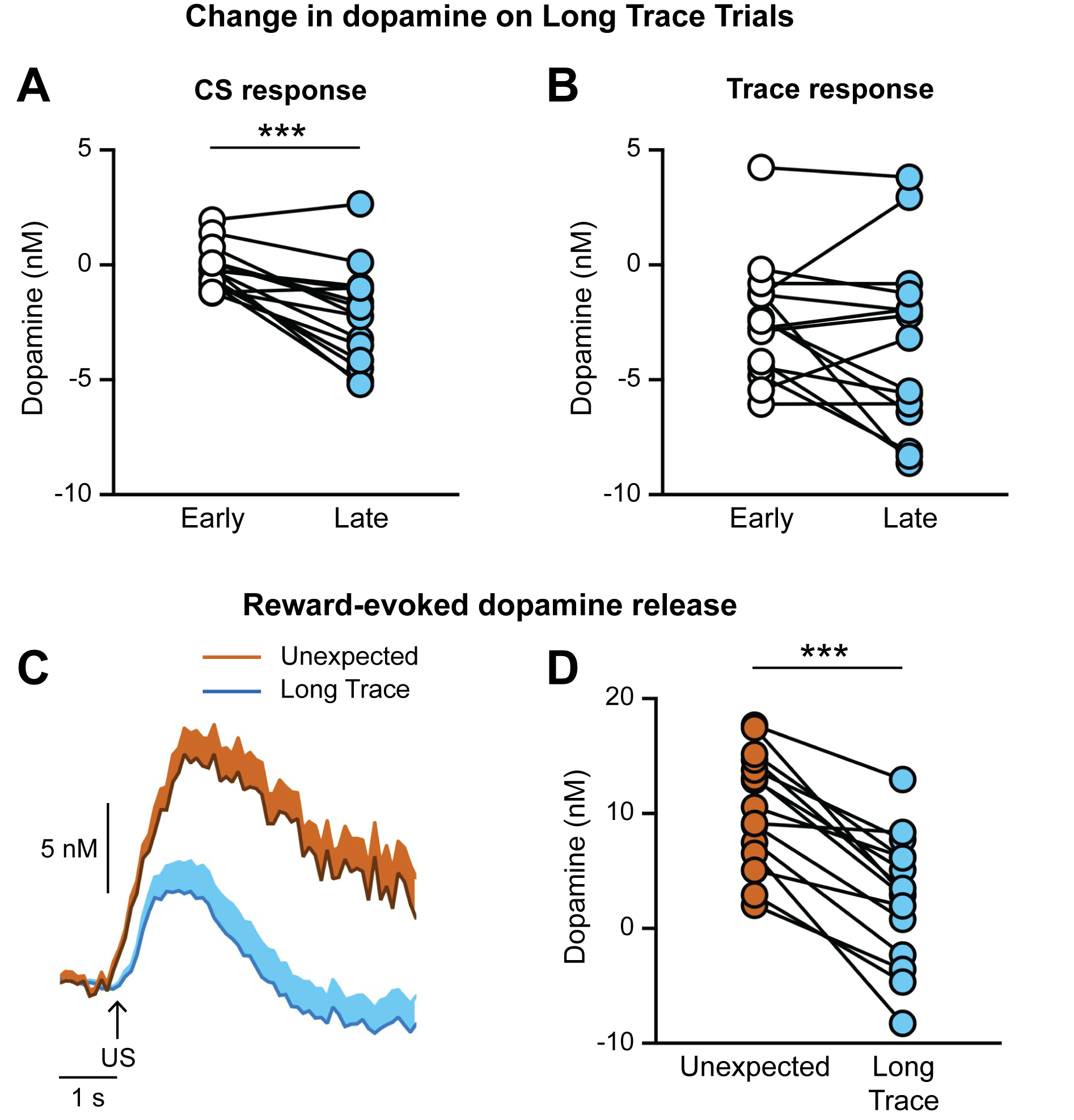

Supplement: Figure 2-1 — Additional analyses of NAc dopamine signals. (A,B) Change in dopamine on Long Trace trials during the CS period (A) and the first 5 s of the trace period (B). Values were calculated by examining the average dopamine signal during the first 1 s and last 1 s of the epoch. Dopamine levels significantly decreased during the CS presentation (A, paired t-test: t14 = 4.9, *** p = 0.0002), but had no further change during the initial trace period (B, paired t-test: t14 = 1.5, p = 0.14). (C,D) US-evoked dopamine release on Long Trace trials is significantly smaller than the dopamine response to an unexpected food pelleted delivered outside of the Pavlovian conditioning sessions (paired t-test: t14 = 7.7, *** p < 0.0001). Download Figure 2-1, TIF file. [file eneuro-12-ENEURO.0016-25.2025-s003.tif]
